# Participatory approaches and methods in gender equality and gender-based violence research with refugees and internally displaced populations: a scoping review

**DOI:** 10.1186/s13031-023-00554-5

**Published:** 2023-12-08

**Authors:** Michelle Lokot, Erin Hartman, Iram Hashmi

**Affiliations:** London School of Hygiene and Tropical Medicine, London, UK

**Keywords:** Refugee, Internally displaced person, Participation, Research, Gender, Gender-based violence

## Abstract

Using participatory approaches or methods are often positioned as a strategy to tackle power hierarchies in research. Despite momentum on decolonising aid, humanitarian actors have struggled to describe what ‘participation’ of refugees and internally displaced persons (IDPs) means in practice. Efforts to promote refugee and IDP participation can be tokenistic. However, it is not clear if and how these critiques apply to gender-based violence (GBV) and gender equality—topics that often innately include power analysis and seek to tackle inequalities. This scoping review sought to explore how refugee and IDP participation is conceptualised within research on GBV and gender equality. We found that participatory methods and approaches are not always clearly described. We suggest that future research should articulate more clearly what constitutes participation, consider incorporating feminist research methods which have been used outside humanitarian settings, take more intentional steps to engage refugees and IDPs, ensure compensation for their participation, and include more explicit reflection and strategies to address power imbalances.

## Introduction

Within research, ‘participation’ has often been understood as the process of directly involving people who are affected by a particular issue, in the process of research [1]. Humanitarian actors, including international non-governmental organisations (NGOs), UN actors and local NGOs assert the importance of participation of populations affected by crises—refugees and internally displaced persons (IDPs)—in humanitarian activities. The Humanitarian Accountability Partnership’s (HAP) 2013 standard—a key humanitarian guideline—positions participation as vital to humanitarian accountability. HAP defines participation as: ‘listening and responding to feedback from crisis-affected people when planning, implementing, monitoring and evaluating programmes, and making sure that crisis-affected people understand and agree with the proposed humanitarian action and are aware of its implications’ [2].

The concept of ‘participatory research’ is sometimes used when discussing how to enhance participation in research. Caroline Lenette and colleagues suggest that when talking about participatory research, there is a difference between taking a ‘holistic approach’ within a broader ‘participatory paradigm’ and using methods identified as ‘participatory’ such as PhotoVoice, that is, a difference between methodology (or approach) and method [1]. In this paper, we use their framing of approach versus method to distinguish between efforts to embed participatory strategies within research holistically, in contrast with using participatory research methods, while also recognising that both of these framings may co-exist within a research project. Examples of taking a holistic approach include ‘community-based participatory research’ (CBPR) and Participatory Action Research (PAR). CBPR has been used to ensure refugees/IDPs are involved at every stage of the research process, and focuses on ensuring that research practices address unequal power hierarchies and adhere to ethical principles [3, 4]. PAR also represents a research paradigm/approach focused on working with populations affected by an issue to generate momentum for change. Scholars urge that care is taken with implementing PAR, because of the risk of creating false hope that action will be taken based on the research [5]. Research may be labelled as using PAR without real meaning: ‘The trend of putting the terms “participatory” and “action” before “research” has led to co-option: not every project labelled PAR is “participatory” research…’ [6]. Different to this holistic approach, certain research methods are often associated with being participatory, for example PhotoVoice, theatre or arts-based methods. Scholars have observed the ‘glorification’ of arts-based methods, which may be implemented blindly because they are seen as participatory, creative and innovative—without consideration of the relevance of these methods for affected populations [7].

The concept of participation has become more common within the humanitarian sector as a result of how it has been operationalised within international development, including through the work of practitioners such as Robert Chambers [8]. In the development sector, participation was a means of shifting power back to communities, for example, through approaches like ‘participatory rural appraisal’ [9]. Some have critiqued these efforts, labelling them unsuccessful in shifting power dynamics within international development [10]. Others point to external shifts that have decreased the focus on hearing directly from affected populations, including mandates from donors that development and humanitarian actors deliver impact and value for money [11]. Despite participation sometimes being connected to improving efficiency [12], in humanitarian settings the capacity to be participatory is often pitted against the urgency of responding to crises. For example, taking the time to listen to refugees/IDPs is seen as too challenging with the limited funding offered by short-term emergency projects [13]. There may also be a distinction between listening to refugees/IDPs and actively involving them in design and analysis of research, especially when listening occurs in an extractive way [7]. Further complicating matters, the term ‘participation’ is sometimes used interchangeably with other terms, such as inclusion, engagement and involvement [14, 15]. Outside of international development and humanitarian action, participatory approaches and methods are recognised as holding important potential for shifting power [1, 16], transforming knowledge production [17], increasing equity [18, 19], ensuring marginalised populations are reached [20–22], and enabling innovative research practice and methods [21, 23, 24].

Humanitarian actors have sought to create processes to enhance the participation of refugees and IDPs within humanitarian activities, including research. Research with refugees and IDPs may be conducted by academic or humanitarian actors, and may include baselines, assessments, evaluations and specific research studies. Within such research, efforts to promote participation may include training refugees and IDPs to collect data themselves, consulting them on their needs, and ensuring that they share their perspectives during evaluations. Humanitarian actors invoke the concept of participation to varying degrees: in instrumental ways to achieve better outcomes, and in practical ways such as through their relationships with refugees and IDPs [25].

Efforts to enhance refugee/IDP participation in research have been criticised for being tokenistic, stemming from the concept of participation being ‘externally imposed’ [15]. Involvement of refugees within research has been described as ‘exploitative’, whereby refugees are treated as merely sources of data rather than as individuals [26]. Conflict-affected populations have expressed frustration with being convened for ‘consultations’ when humanitarian actors have already made decisions about their needs and identified solutions [27]. Humanitarian actors have also been criticised for only promoting women’s participation to improve efficiency [9] and for failing to recognise how gender, age, ethnicity, economic status and other power hierarchies might constrain participation in humanitarian settings [28], which increases the influence of power-holders like refugee elites [29]. These critiques are not necessarily new, but demonstrate there is lack of clarity on what it means for research to reflect ‘refugee voices’ [30]. Efforts to be ‘participatory’ often lack clarity on what this means [31].

Critiques of poor implementation of participation have not specifically been applied to gender equality research. Gender equality research—which includes research on gender-based violence (GBV)—often involves consideration of power dynamics, thus often positions participation as a pre-cursor for gender equality [9]. Participatory research and feminist research share common goals of empowering marginalised populations [1]. Understanding how participation occurs within research on gender equality may provide important lessons for how participation is being used in research which already uses power as a key lens. For example, while not among refugees and IDPs, recent examples of feminist participatory research with other populations have considerably advanced scholarship through piloting new methods such as body mapping to understand inequity [32], digital mapping to conceptualise street harassment [33] and participatory video to provide new insights on gender inequalities [34]. Feminists have provided critical new insights for participatory research, such as through emphasising not just women’s voices but also their silences during the research process [35], and reframing ethics from women’s perspective [36]. Evaluation practice has also been transformed through use of feminist participatory action research approaches that position evaluation participants as co-researchers, challenging the power dynamics often built into evaluation processes [37, 38]. Feminist participatory research has provided particular insights for research on violence, including agenda-setting on the use of trauma-informed approaches [39, 40], integrating feminist principles into quantitative studies on violence [41] and using indigenous feminist approaches to reframe women’s safety [42]. Feminist research approaches and methods continue to push the boundaries of what it means to be ‘participatory’ in diverse settings [43].

This scoping review explores academic and grey literature on gender equality and GBV among refugees and IDPs which describes itself as ‘participatory’. Specifically, the objectives of this review were to: (1) describe the contexts, approaches and methods used in gender and GBV research with refugees and IDPs; (2) outline the rationale and impacts of promoting refugee/IDP participation in research; (3) describe how refugee/IDP participation is conceptualised, including how participatory approaches and methods are used in research.

## Methods

We followed the Preferred Reporting Items for Systematic reviews and Meta-Analyses extension for Scoping Reviews (PRISMA-ScR) to conduct and report on this scoping review [44]. We conducted a scoping review rather than a systematic review to recognise that the body of evidence on refugee/IDP participation in research on gender and GBV is still emerging, and to acknowledge that we must understand how the literature defines participation, what methods are used and what evidence currently exists on the topic. Since we were focused on understanding the concept of participation rather than addressing effectiveness or appropriateness [45], a scoping review was deemed the best approach. In line with Chang’s approach for scoping reviews [46], instead of summarising and assessing the quality of evidence, we explored the literature, identified key definitions and themes and identified the type and nature of evidence available.

### Search strategy

We searched five academic databases (Medline, PsycINFO, Academic Search Complete, Web of Science and Scopus) in February 2022. The database searches included search terms related to three main concepts: (1) gender equality and GBV, (2) refugees/IDPs, and (3) participation. Table 1 outlines the key search terms used for each database.

**Table 1:** Key search terms for each database

Academic databases	Abstract and title search terms*
Medline, PsycINFO, Academic Search Complete, Web of Science and Scopus	*TERM ONE: gender equality and GBV* “gender equality” OR “gender inequality” OR “gender equit*” OR “gender inequit*” OR “gender” OR “masculinit*” OR “femininity*” OR “gender norm*” OR “power dynamic*” OR “gender dynamic*” OR “gender role*” OR “women’s empowerment” OR “empowerment of women” OR “empowerment of girls” OR “girls’ empowerment” OR “patriarch*” OR “GBV” OR “gender-based violence” OR “violence against women” OR “sexual violence” OR “physical violence” OR “emotional violence” OR “psychological violence” OR “verbal abuse” OR “intimate partner violence” OR “domestic violence” OR “abuse” OR “femicide” OR “feminicide” OR “human trafficking” OR “trafficking of persons” OR “partner violence” OR “abuse of women” OR “wife abuse” OR “abuse of wives” OR “wife battering” OR “battering of wives” OR “battering of women” OR “spouse abuse” OR “family violence” OR “murdering of women” OR “homicides of women” OR “honour killing” OR “honor killing” OR “acid attack*” OR “acid throwing” OR “sex selective abortion” OR “missing women” OR “missing girls” OR “widow burning” OR “stoning of women” OR “rape” OR “sexual assault” OR “sexual harassment” OR “coerced sex” OR “unwanted sex” OR “unwanted fondling” OR “unwanted touching” OR “harmful traditional practices” OR “FGM” OR “FGC” OR “female genital mutilation” OR “female genital cutting” OR “child marriage” OR “forced marriage” OR “early marriage” OR “sexual exploitation” OR “forced prostitution” OR “sexual slavery” *TERM TWO: refugee/IDP* “refugee*” OR “internally displaced person*” OR “IDP” OR “asylum-seeker*” *TERM THREE: participation* “participat*” OR “engag*” OR “inclusi*” OR “involv*” OR “take part” OR “took part”

*MeSH terms were used for Medline and PsycINFO. MeSH terms were not effective for the other databases

We supplemented the academic database search by searching Google and Google Scholar using the following search strings: “refugee participation” AND gender; “refugee participation” AND gender-based violence; refugee AND gender AND participatory research; displaced AND gender AND participatory research. We limited results for Google and Google Scholar to the first 200 hits per search and cleared browsing data after each search. All searches were conducted without signing into Google to prevent tailoring of results by location and search history [47]. We searched institutional websites of organisations working on gender, GBV and refugee/IDP research, specifically: UNFPA, UN Women, UNHCR, Women’s Refugee Commission and International Center for Research on Women. We also asked practitioners and researchers in this field to send articles that may fulfill inclusion criteria through the Sexual Violence Research Initiative and Forced Migration mailing lists. We hand-searched the reference lists of included papers to identify additional records for inclusion. In order to prevent publication bias and avoid excluding knowledge produced by non-academic actors, we intentionally searched sources outside of academic databases [48].

### Inclusion and exclusion criteria

Articles in English from any time period and country involving empirical research with refugees/IDPs on gender equality or GBV were included. We included high-income settings where refugees are resettled like the United States, Australia, European countries and Canada, recognising firstly that there has been considerable investment in participatory research and emerging scholarship on what it means to be ‘participatory’ from these settings; and secondly that the challenges in active conflict and humanitarian settings would likely prevent participatory research from occurring.

Screening occurred in two stages using Covidence. First, we screened titles and abstracts, excluding non-empirical research, studies unrelated to gender equality or GBV and studies that collected data only amongst host populations or amongst practitioners, rather than refugee/IDP populations,were also excluded. During the full-text review, we narrowed our criteria to search full texts for descriptions of efforts to promote participation of refugees/IDPs. Studies that did not incorporate this term or various forms of it (e.g. ‘participatory’ and ‘involvement’) were excluded. Where multiple records by the same author existed for the same research, only the earliest record was included. During the title/abstract screening and full-text review process, all articles were double-screened with regular meetings held between the three researchers to reach consensus. The first author reviewed all articles at both stages. Table 2 outlines the inclusion and exclusion criteria.

**Table 2:** Inclusion and exclusion criteria

INCLUSION	EXCLUSION
Empirical research studies	Editorials, letters, commentaries, literature reviews (including systematic reviews), conference proceedings, opinion pieces, books, book chapters, theses
Topic of research is gender equality, or gender-based violence	Topic of research is something other than gender equality or gender-based violence
Refugees and internally displaced populations are research participants in the study	Only host populations are research participants
Refugees and IDPs are living in any country (to capture research with resettled refugee populations)	No exclusions
Studies conducted over any time period	No exclusions

### Data analysis

For each included article, we extracted information on: (a) study design (country, type of population, nationality of refugees/IDPs, sample size, research methods), (b) type of gender equality or GBV issue, and (c) participation (level of focus on participation, definitions of participation, rationale for participatory approach, recommendations for future participatory research, impacts of participation). For population type, we classified based on how the populations were described in the study, rather than using legal definitions of refugees, IDPs, migrants or asylum seekers. We defined ‘gender equality’ using UN Women’s definition as ‘equal rights, responsibilities and opportunities of women and men and girls and boys’ [49]. We initially classified studies on three levels according to the degree to which the participation was a focus: (1) low: participation in research is mentioned in passing/without further discussion or explanation, (2) medium: participation in research is referenced only in the methods section, or (3) high: participation in research is referenced in the methods section as well as throughout the paper.

Data was extracted using Covidence. Each article was extracted by two authors, with the first author extracting every article. We analysed extracted data to identify: whether and how participation was defined and to what extent it was a focus; the types of methods and strategies used to ensure participation of refugees/IDPs in research; the rationale for promoting participation, including how power dynamics were framed; the impacts of participation; and recommendations for improving participation.

### Limitations

Our review has a few limitations. Firstly, due to time and staffing constraints, we only searched for a few key concepts related to participation in academic databases, rather than specifically searching for methods or methodologies commonly identified as participatory. This may have limited the studies that were identified in the database search. Secondly, our review is limited by whatever content authors chose to include in their papers, which may not have been fully representative of the holistic approach taken to participation or to the participatory methods used. Authors may have been constrained by their journal requirements, and may not have been able to include the full level of detail. In at least two cases [50, 51], methods sections were shorter because the authors subsequently published a solely methods-focused paper—which fell outside the scope of our review. As with any review, our analysis is confined to what authors describe, which may only be a snapshot of what occurred in their research. Finally, our ranking approach was not a straight-forward process and often required judgments be made about the level of content on participation included by authors. While we made decisions about rankings together, it is possible that the lines between categories are more blurred.

## Findings

### Final sample

Out of 2641 results from five academic databases, 1092 were duplicates, resulting in 1549 unique records being screened.

Alongside the academic database records, 88 additional records were identified and screened from Google Scholar (n = 50), Google (n = 26), institutional websites (n = 1), practitioners (n = 7) and through hand-searching references from included papers (n = 4). After screening, 35 of these were included in the full-text review and 8 of these were deemed eligible.

**Fig. 1:**
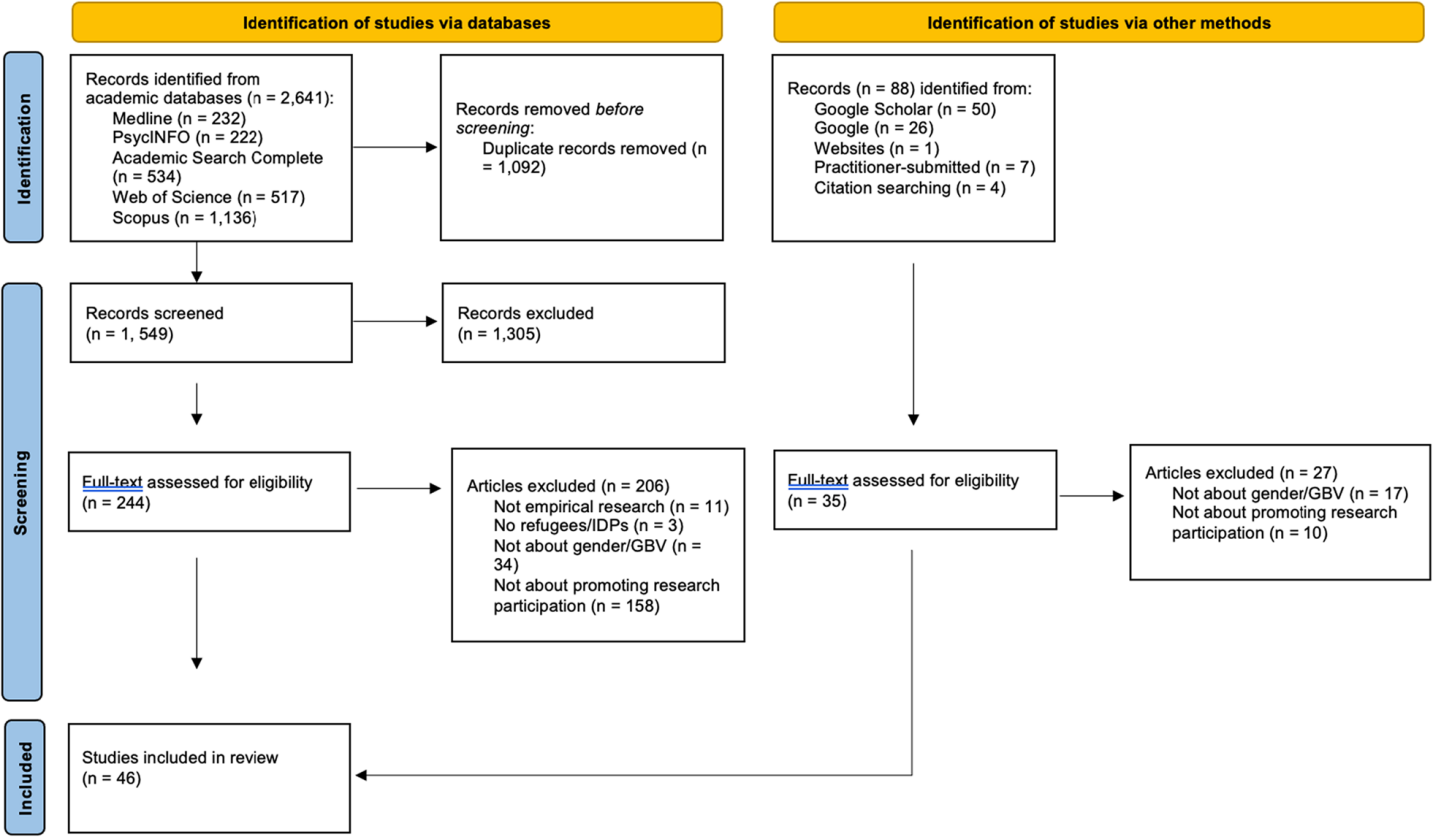
Adapted PRISMA framework

We assessed 244 full-text papers from academic databases for eligibility. Of these, 206 studies (84%) were excluded due to not being empirical research (n = 11), not including refugees/IDPs (n = 3), not being about gender/GBV (n = 34), or not mentioning referencing being participatory in approach or using a participatory method (n = 158). Among studies from other sources, 27 studies (77%) were excluded due to not being about gender/GBV (n = 17) or not being about promoting participation (n = 10). In total, 46 studies were included, specifically 38 from academic databases and 8 studies from other sources. Figure 1 outlines the scoping review process at different stages using an adapted PRISMA framework.

### Study types and design

Out of the 46 included studies, 39 adopted a qualitative design and the remaining seven employed quantitative (n = 3) and mixed methods (n = 4). The qualitative studies utilized various methods, including semi-structured interviews, focus group discussions (FGD), ‘participatory group discussions’, and participatory mapping and ranking approaches. In total, eight studies used photography as a research method, with three explicitly mentioning using ‘PhotoVoice’ and the rest adopting a participatory and ethnographic photographic approach. Quantitative studies mainly used surveys, whereas mixed method studies employed interviews and FGDs in addition to surveys. Table 3 shows the characteristics of the studies in this review.

**Table 3:** Key characteristics of included studies

Reference	Title	Country	Population	Study design	Study methods
Affleck, W. et al. [52]	“If one does not fulfil his duties, he must not be a man”: masculinity, mental health and resilience amongst Sri Lankan Tamil refugee men in Canada	Sri Lanka and Canada	Refugees & IDPs	Qualitative	Interviews
Ager et al. [53]	Local constructions of gender-based violence amongst IDPs in northern Uganda: analysis of archival data collected using a gender- and age-segmented participatory ranking methodology	Uganda	IDPs	Qualitative; other	FGDs with participatory ranking approaches
Al Akash and Chalmiers [54]	Early marriage among Syrian refugees in Jordan: exploring contested meanings through ethnography	Jordan	Refugees, Practitioners & Other stakeholders	Qualitative	Interviews, participatory action research (PAR) meetings, participant observation
Dantas et al. [55]	Empowerment and health promotion of refugee women: the Photovoice project	Australia	Refugees	Qualitative; other	PhotoVoice and interviews
Edstrom and Dolan [56]	Breaking the spell of silence: Collective healing as activism amongst refugee male survivors of sexual violence in Uganda	Uganda	Refugees	Qualitative; other	Interviews, group work, participatory video testimonies, participatory film production
Ellis [57]	Discrimination and mental health among Somali refugee adolescents: the role of acculturation and gender	United States	Refugees	Mixed Methods	Interviews, use of surveys/scales
Elnakib et al. [58]	Drivers and consequences of child marriage in a context of protracted displacement: a qualitative study among Syrian refugees in Egypt	Egypt	Refugees, Practitioners & Other stakeholders	Qualitative; other	Interviews, FGDs with photo elicitation and participative ranking approach
Fincham [59]	Constructions, contradictions and reconfigurations of 'Manhood' among youth in Palestinian camps in Lebanon	Lebanon	Refugees	Qualitative; Other	Participant observation, semi-structured inter- views, focus groups and Participatory Learning and Action (PLA) approaches, such as taxonomies, sorting and ranking techniques, Venn diagrams, art and role-play
Fineran and Kohli [60]	Muslim refugee women's perspectives on intimate partner violence	United States	Refugees & Asylum Seekers	Qualitative	In-depth interviews
Fisher [61]	Changed and changing gender and family roles and domestic violence in African refugee background communities post-settlement in Perth, Australia	Australia	Refugees	Qualitative	Interviews with refugees, FGDs with staff
Fobear [62]	Nesting Bodies: Exploration of the body and embodiment in LGBT refugee oral history and participatory photography	Canada	Refugees	Qualitative; other	Participatory photography, oral history
Gibb [63]	The evacuation camp as paradoxical space for women	Philippines	IDPs, Practitioners & Other stakeholders	Qualitative; other	Interviews, FGDs, participatory video, non-participant observations counter-mapping
Green and Latifi [64]	No one smiles at me: The double displacement of Iranian migrant men as refugees who use drugs in Australia	Australia	Refugees	Qualitative	FGDs and interviews
Guerin et al. [65]	Advocacy as a means to an end: Assisting refugee women to take control of their reproductive health needs	Australia and New Zealand	Refugees	Qualitative	Interviews, FGDs, participant observation
Gustafson and Iluebbey [66]	'Traditional discipline' or domestic violence": participatory action research with a Sudanese refugee community	United States	Refugees	Qualitative	Interviews, FGDs, participant observation
Holle, F. et al. [67]	Exilic (Art) narratives of queer refugees challenging dominant hegemonies	The Netherlands	Refugees and Asylum Seekers	Qualitative; other	Biographical interviews, arts-based projects
Johnson-Agbakwu et al. [68]	Perceptions of obstetrical interventions and female genital cutting: insights of men in a Somali refugee community	United States	Refugees	Qualitative	Interviews, FGDs
Johnson et al. [69]	Building community-based participatory research partnerships with a Somali refugee community	United States	Refugees & Practitioners	Mixed Methods	Surveys, interviews, FGDs with video elicitation
Keygnaert et al. [70]	Sexual violence and sub-Saharan migrants in Morocco: a community-based participatory assessment using respondent driven sampling	Morocco	Refugees & Migrants	Qualitative	Interviews
Keygnaert et al. [71]	Sexual and gender-based violence in the European asylum and reception sector: a perpetuum mobile?	Belgium, Greece, Hungary, Ireland, Malta, The Netherlands, Portugal & Spain	Refugees, Undocumented migrants & Asylum Seekers	Quantitative	Surveys
Lee and Brotman [72]	Identity, refugeeness, belonging: Experiences of sexual minority refugees in Canada	Canada	Refugees	Qualitative	Interviews
Lenette et al. [1, 23, 73]	Mothers & Daughters: Redefining cultural continuity through South Sudanese women’s artistic practices	Australia	Refugees	Qualitative	Unstructured group discussions during arts-based workshops, participant observation
Lokot [28]	The space between us: feminist values and humanitarian power dynamics in research with refugees	Jordan	Refugees & Practitioners	Qualitative; other	Participatory photography, semi-structured interviews, life-story interviews and participant observation
McMorrow and Saksena [74]	Voices and views of Congolese refugee women: A qualitative exploration to inform health promotion and reduce inequities	United States	Refugees	Qualitative; other	Photovoice, interviews
Mehta et al. [75]	Learning from UJAMBO: Perspectives on gynecologic care in African immigrant and refugee women in Boston, Massachusetts	United States	Refugees & Immigrants	Qualitative	FGDs
Mulé [76]	Mental health issues and needs of LGBTQ + asylum seekers, refugee claimants and refugees in Toronto, Canada	Canada	Refugees & Asylum Seekers	Qualitative	FGDs
Murray et al. [77]	Between 'here' and 'there': family violence against immigrant and refugee women in urban and rural Southern Australia	Australia	Refugees, Practitioners & Immigrants	Qualitative	In-depth interviews and with key informants
Pangcoga and Gambir [78]	Our voices, our future: understanding risks and adaptive capacities to prevent and respond to child marriage in the Bangsamoro Autonomous Region in Muslim Mindanao (BARMM)	Philippines	IDPs & Practitioners	Qualitative; other	Interviews with practitioners, SenseMaker, participatory group activities
Potts et al. [51]	Empowered Aid: Transforming gender and power dynamics in the delivery of humanitarian aid. Participatory action research with refugee women & girls to better prevent sexual exploitation & abuse—Lebanon Results Report	Lebanon	Refugees	Qualitative	Participatory group discussions and interviews
Potts et al. [50]	Empowered Aid: Transforming gender and power dynamics in the delivery of humanitarian aid. Participatory action research with refugee women & girls to better prevent sexual exploitation & abuse—Uganda Results Report	Uganda	Refugees	Qualitative	Participatory group discussions and interviews
Rahamtalla and Saeed [79]	Gender analysis of the impacts of displacement on Western Sudanese migrants in Khartoum State, Sudan	Sudan	IDPs	Quantitative	Survey
Rees and Pease [80]	Domestic violence in refugee families in Australia: rethinking settlement policy and practice	Australia	Refugees	Qualitative	Interviews, FGDs
Rezaian et al. [81]	Gendered spaces and educational expectations: the case of the former refugee camp “Elliniko” in Athens	Greece	Refugees	Qualitative	Observations, classroom interactions, language portraits, semi-structured interviews, focus groups, diaries
Ritterbusch [82]	Mobilities at gunpoint: The geographies of (Im)mobility of transgender sex workers in Colombia	Colombia	IDPs	Qualitative; other	Interviews, auto-photography, mapping
Rothkegel et al. [83]	Evaluation of UNHCR’s efforts to prevent and respond to sexual and gender-based violence in situations of forced displacement	Tanzania, DRC, Yemen, Nepal & Georgia	Refugees, IDPs & Practitioners	Qualitative	Interviews and participatory workshops
Schulte and Rizvi [84]	In search of safety and solutions: Somali refugee adolescent girls at Sheder and Aw Barre Camps, Ethiopia	Ethiopia	Refugee & Practitioners	Qualitative; other	Interviews and FGDs with participatory safety mapping
Scott [85, 86]	An assessment of gender inequitable norms and gender-based violence in South Sudan: a community-based participatory research approach	South Sudan	IDPs	Quantitative	Survey
Shanahan and Vaele [87]	How mothers mediate the social integration of their children conceived of forced marriage within the Lord’s Resistance Army	Uganda	IDPs	Qualitative; other	Interviews and photo-ethnography
Simbandumwe et al. [88]	Family violence prevention programs in immigrant communities: Perspectives of immigrant men	Canada	Refugees & Immigrants	Qualitative	Interviews; FGDs
Stark et al. [89]	Disclosure bias for group versus individual reporting of violence amongst conflict-affected adolescent girls in DRC and Ethiopia	Ethiopia & DRC	Refugees & IDPs	Mixed Methods; other	Survey, FGD, mapping activity
Sullivan et al. [90]	"For us it is like living in the dark": Ethiopian women's experiences with domestic violence	United States	Refugees & Immigrants	Qualitative	FGDs
Tanabe et al. [91]	Intersecting sexual and reproductive health and disability in humanitarian settings: risks, needs, and capacities of refugees with disabilities in Kenya, Nepal, and Uganda	Kenya, Nepal, Uganda	Refugees	Qualitative; other	Body mapping, timelines, sorting, interviews, FGDs
Thompson [92]	Exploring gender and culture with Khmer refugee women: reflections on participatory feminist research	United States	Refugees	Qualitative	FGDs; Participant observation; Dream narratives, interpretations and amplifications; Translations of original Khimer myths
Vloeberghs et al. [93]	Coping and chronic psychosocial consequences of female genital mutilation in the Netherlands	The Netherlands	Refugees	Mixed Methods	Survey, interviews
Weber [94, 95]	Participatory visual research with displaced persons: ‘Listening’ to post-conflict experiences through the visual	Colombia	IDPs	Qualitative; other	Photovoice, participant observation, interviews, focus groups
Whiting-Collins et al. [96]	Fostering protective assets among Syrian refugee girls who experience child marriage: Findings from a formative program evaluation	Lebanon	Refugees	Qualitative	Interviews; FGDs

### Study settings, populations and funders

Included studies were conducted in 29 countries, with the most studies conducted in the United States (n = 8), followed by Australia (n = 7) and Uganda (n = 5). Most studies were conducted in only one country (n = 40) only, while a smaller number were conducted in three countries (n = 2) or two countries (n = 2). One study was conducted in 8 countries and another in 5 countries. According to geographical region, North America (n = 12), Australia/Asia (n = 13) and sub-Saharan Africa (n = 13) were the most (and equally) represented, followed by Europe/Caucuses (n = 12), and the Middle East and North Africa (n = 8). Only two studies were conducted in South America, both in Colombia.

Overall, close to half the studies (n = 21) collected data solely from refugees. A further 17 studies included some combination of refugees with other populations such as IDPs, (n = 2), practitioners (n = 3), practitioners and other stakeholders (n = 2), migrants (n = 1), asylum seekers (n = 3), undocumented migrants and asylum seekers (n = 1), immigrants (n = 3), immigrants and practitioners (n = 1), and IDPs and practitioners (n = 1). In total 6 studies focused solely on IDP populations, while a further 2 focused on IDPs and practitioners (n = 1) and IDPs, practitioners and other stakeholders (n = 1).

### Study methods

Studies employed several different, and sometimes mixed, research methods.

Qualitative methods were most commonly used (93% of included studies used qualitative methods alone or in combination with other methods), and were predominantly structured as interviews or focus group discussions. Interviews were conducted with refugees/IDPs or other community-based actors and took the form of in-depth, semi-structured or biographical interviews. Focus group discussions were formal and informal, stratified by age and gender, or designed as workshops or anecdote circles. Researchers employed varied—and creative and participatory—methods within such interviews and focus group discussions to collect data and learn about the nuances of refugee/IDPs lives and experiences. These techniques included: storytelling, oral histories, and vignettes, safety, community, dream, and body mapping, free listing, timelining, ranking, sorting, and venn-diagramming, art making, document analysis, photo-elicitation, diaries and role play. Studies also used qualitative methods such as observations and methodologies such as ethnographies.

Studies also employed PhotoVoice (or derivatives of participant or auto-photography) and artistic co-creation. Through the taking of photos and their presentation and discussion, photo-based methods enable community strengths, issues, and concerns to be documented and can promote critical dialogue [55]. Types of artistic co-creation included song, written tests, deejay sets, ‘Grindr poetry’, video poetry, performance, drag, and graphic design [67].

Researchers also utilised quantitative or mixed-qualitative and quantitative methods for data collection. Three studies used quantitative methods alone, including a knowledge, attitudes and practices survey, a randomised household survey with ‘heads of households’ [79] and an attitude survey incorporating the ‘Gender Equitable Men’ scale [85]. Two of these quantitative studies described their participatory approach as involving the creation of advisory groups consisting of refugees who were involved in decision-making about the research [71, 85], however the third mentioned using a ‘participatory approach’ and ‘participatory method’ without further explanation [79]. Further, in this third study, only sampling household heads is limiting as this often results in over-representation of men, limiting women’s participation as research participants. Mixed methods included prioritization exercises (with numerical rankings) and the use of the ‘Sensemaker’ method, which documents micro-narratives of refugees/IDPs lived experiences and, then from these narratives, using a signification framework, participants then create their own set of questions to analyze such narratives [78].

As will be discussed in later sections, some of these methods were explicitly framed as being participatory. These research methods are distinct from the broader participatory approaches employed.

### Gender and GBV focus

In total, 68% of included studies (n = 32) focused on GBV. This included 14 studies that focused solely on GBV, and 18 studies which looked at GBV along with other themes specifically: GBV and adolescent girls (n = 4), GBV and LGBTQIA + (n = 4), GBV and sexual and reproductive health (n = 2), and various combinations of GBV with other topics including economic development, maternal and child health, economic development, division of labour/gender roles, decision-making/leadership and masculinities.

The remaining 32% of included studies (n = 15) focused on topics related to gender equality more broadly without discussing GBV. These topics included LGBTQIA + (n = 4), division of labour/gender roles (n = 2), sexual and reproductive health (n = 2), masculinities (n = 1), and various combinations of division of labour/gender roles with other topics (n = 6). The greater proportion of studies focused on GBV rather than gender equality more broadly may reflect the fact that researching GBV requires greater sensitivity and care (which participatory approaches and methods may help with). For included papers focusing on humanitarian settings (rather than high-income countries hosting refugees), the emphasis on gender equality may also reflect the greater focus within the humanitarian sector on GBV compared to other gender-related issues.

### Definitions of participation and ‘participatory’ research

Across all included studies, no definition of the core concept of ‘participation’ was discussed, despite recognition that participation is important. Existing frameworks and definitions were not referenced in these studies.

However, included studies do describe or define different participatory approaches to research. For example, Lenette and colleagues [73] describe participatory research as research that ‘begins from a social, ethical and moral commitment not to treat people as objects of research but rather, to recognise and value the diverse experiences and knowledges of all those involved (…) Participatory research is often seen as a method that promotes cultural continuity and values gender-specific standpoints’ (757). Feminist participatory research is described by Thompson [92] as ‘a conscious break with research programs grounded in empiricism (…) Feminist participatory research, then, is not just neutral on the topic of women. It is instead openly committed to a diverse range of women's experiences and women's struggles. It is guided by feminist critiques of science and employs methods that preserve women's experiences in context’ (31).

The concept of ‘Participatory Action Research’ (PAR) was also described in several studies, with a focus on principles of PAR [50–52, 60, 65]. Community-based participatory research (CBPR) principles were also discussed in a few studies [64, 68, 69]. Other concepts that were described were PhotoVoice [74], action research [66] and ‘community participatory methodology’ [57].

### Rationale for promoting refugee/IDP participation

Reviewed papers provided several rationales for promoting participation of refugees/IDPs in their research including: their identity as a refugee/IDP, their gender, and their position within power hierarchies. For example, various papers (n = 9) voiced that the experiences and qualities that are intrinsic to refugee/IDP status mandated their active participation in research. With a consensus that there is an overall lack of attention to this population [64], coupled with their rapidly increasing numbers [74], authors believed it was especially important to include those with “local, individual and marginalized viewpoints” [59] that are often outside of traditional “Western” research [68, 69], in order to capture a holistic view of their lives [94]. Authors also viewed their participation as an empowering process, which could counter act often romanticized perceptions and representations of their lives, such as that they are all traumatized [95]. Participatory research was also positioned as responding to the fact that research with refugees does not use strengths-based approaches [55]. Thompson believed participation—via the recall and collection of their stories—could help participants to reconstruct their lives [92].

Further, several reviewed papers (n = 8) cited feminist theory as a rationale for promoting participation amongst research with refugees/IDPs [28, 55, 67, 70, 74, 77, 92, 94]. Most referred to encouraging women and girls to join the research process. However, one paper purposely included adolescent boys so to understand their perspectives on issues around gender inequality and marriage [78] and a few purposely included LGBTQIA + refugees/IDPs (n = 2). Overall, rationales for including women and girls were two-fold. First, they either conceptualized knowledge as a (feminist) process of emancipation and social change [95], and thus, included women and girls to address gender stereotypes that persist in research [74]. For example, they recognized that women and girls are less likely to participate in mixed-gendered research spaces and that their contributions to knowledge are often viewed as less valuable [95]. Secondly, the rationale used for including women and girls was in order to ensure that research recommendations would be centred on their specific needs and experiences, for example, to ensure that their specific safety concerns would be included.

Moreover, many studies (n = 10) sought to include refugees/IDPs within the research process to address the power imbalances that are often present within research and ensure more democratic equitable research. This often included descriptions of how power dynamics can make research ‘exploitative’ [95]. In Pangcoga and Gambir’s study, the Sensemaker method made the research more ‘democratic’, enabling participants voices to be centred while addressing power imbalances [78], while others identified how their choice of methods such as participatory photography, visual methods and production of artistic outputs helped to reduce power dynamics [28, 67, 94]. In both ‘Empowered Aid’ studies, PAR was stated as a means of recognising and tackling power imbalances [50, 51]. Other studies also took a holistic approach to being participatory through strategies such as asking open-ended questions [28, 73], reflecting on power and positionality [28, 92, 94], spending more time with refugees and thinking about how best to represent their lives [28, 72, 94]. Studies acknowledged that it was challenging to fully address power imbalances [95].

### Level of focus on participation

As part of the extraction process, we classified included studies according to how authors’ described their study’s focus on on participation. This was driven by our recognition—also discussed in literature—that the concept of participation has often been co-opted by authors [6] when describing their methods, without due consideration to the fidelity and robustness of participation. Firstly, we classified 15% of studies (n = 7) as having ‘low’ content on participation—describing studies where being participatory was mentioned in passing only, without further explanation. We then used the framing by Lenette et al. [1] to contrast the use of a participatory ‘approach’ (i.e. a holistic process made up of multiple strategies to embed participation across the research process), and the use of a participatory ‘method’ (i.e. the use of a specific research method such as PhotoVoice or video). We created three categories to classify the studies that were not categorised as ‘low’: studies that only use participatory method(s), studies that only use a participatory approach, or studies that use both a participatory method and participatory approach. We suggest that simply using a participatory method is not always sufficient to address power hierarchies within research, rather using a more holistic participatory approach encompassing multiple strategies is more helpful.

#### Low

Content classified as ‘low’ (n=7) tended to involve singular references to participation or being participatory without any further explanation [61, 79–81, 86, 96, 97]. For example, using the term ‘participatory qualitative design’ only in the abstract with no additional reference in the text [61], or referring to a ‘participatory approach’ or ‘participatory research’ without further explanation [79, 86, 97].

One study classified as low referred to ‘ethnographic participatory fieldwork’ [81] and listed classroom interactions and language portraits as examples, without explaining these methods further. It is unclear if the methods alone were the reason for using the term ‘participatory’ or if something related to the methodology of the ethnographic fieldwork was participatory. Similarly, another study mentioned ‘participatory FGDs’ and said this involved drawing and poetry, but did not provide further detail on this approach [96], seeming to reflect Ozkul’s [7] critique that arts-based methods are sometimes automatically assumed to be participatory.

Some of these examples may reflect what Cornwall and Brock [98] refer to as ‘buzzwords’. Using the term participation or participatory may invoke positive associations without resulting in refugees or IDPs meaningfully participating in research processes. However, we also recognise that the level of content included to describe participatory approaches and methods are not always reflective of whether studies actually used these approaches. For example, disciplinary styles of writing, journal requirements and feedback from peer reviewers may all result in less (or more) description being added about methods.

#### Studies that only use participatory approaches

In total, 17 studies used a holistic participatory approach in isolation—without also mentioning use of a participatory method. The table below outlines the types of strategies used to enhance participation. We took a broad approach in categorising these studies as taking a participatory approach, recognising that not all practices were explicitly labelled as participatory. For example, one study [72] only mentioned the word ‘participatory’ in passing, yet the practices described in the methods (including having an advisory committee that was connected to the community) align with participatory approaches.

In a few cases, it was not clear if studies also used a participatory method. For example, two studies included community members at each stage of the research as part of the broader participatory approach, but it was unclear if the use of video-elicitation within the FGDs constituted a participatory method [68, 69]. In another case where a feminist participatory approach was described, it was not clear if the use of ‘dream narratives’ may constitute use of a participatory method [92].

#### Studies that only use participatory methods

In total, 11 studies used participatory methods in isolation [53–55, 59, 62, 63, 74, 83, 84, 87, 89]. The methods chosen included participatory photography including participatory mapping [84, 89], PhotoVoice [62] and participatory ranking methodologies [53]. A few studies did not fully explain their use of participatory methods. One study mentioned the use of ‘participatory workshop methods’ multiple times without explaining what this meant [83]. One study used PAR meetings with refugees to gather data [54] and another used ‘participatory learning and action’ (PLA) [59], but neither outlined in detail the PAR and PLA approaches, though Akash & Chalmiers noted that they describe their methodology in another paper [54].

In a few cases, studies were stated as using a participatory approach, however in reality these described methods and were counted within the ten studies above that only talked about methods. In two studies, CBPR was stated as the methodology but only PhotoVoice [55] or only PhotoVoice and interviews [74] were used as the method—and there was no other indication that a broader CBPR approach was taken. Elsewhere PAR was stated as the methodology, but in one study only the use of photo-ethnography as method rather than PAR more broadly was evident based on the paper description [87]. Another study mentioned the use of PAR meetings which were also described as creating space for women ‘to enable women to flexibly tell their own stories of marriage using a life events-narrative approach’ [54]—which sounds less like a participatory method and more like a life history interview.

#### Studies that use both participatory methods and participatory approaches

In total, 11 studies clearly stated the use of both a participatory approach as well as a participatory method [28, 50, 51, 56, 58, 67, 73, 78, 82, 91, 94].

### Strategies used to enhance participation

The most common strategy used within the 27 studies that took a participatory approach was involving participants in design, data collection and analysis (including through an advisory group), which 17 studies mentioned. Other strategies included refugees/IDPs only participating in design/influencing the research agenda (n = 5), refugees/IDPs only participating in analysis/feedback (n = 3), using peer data collectors (n = 4) and providing in-kind or financial compensation for refugees/IDPs who participated (n = 3) (Table 4).

**Table 4:** Key strategies used within studies that took a participatory approach

Key strategy	Study author
Participation of refugees/IDPs throughout the research process: in design, data collection and analysis (including through an advisory group)	Affleck et al. [52], Ellis et al. [57], Fineran and Kohli [60], Green and Latifi [64], Guerin et al. [65], Gustafson and Iluebbey [66], Johnson-Agbakwu et al. [68], Johnson et al. [69], Keygnaert et al. [70], Keygnaert et al. [71], Lee and Brotman [72], Pangcoga and Gambir [78], Potts et al. [51], Potts et al. [50], Ritterbusch [82], Simbandumwe et al. [88], Vloeberghs et al. [93]
Participation of refugees/IDPs only in design/influencing the research agenda	Edström and Dolan [56], Johnson et al. [69], Lenette et al. [73], Lokot [57], Weber [94, 95]
Participation of refugees/IDPs only in analysis/feedback processes	Dantas et al. [55], Edström and Dolan [56], Thompson [92]
Using peer data collectors	Johnson [69], Murray et al. [77], Rezaian et al. [81], Sullivan et al. [90]
Providing in-kind or financial compensation for participants	Gibb [63], Holle et al. [67], Mehta et al. [75]

While this list (which is not mutually-exclusive) represents a helpful indication of the ways in which GBV and gender equality research has sought to promote refugee/IDP participation, it is important to note the challenges in using these strategies which many studies discussed. The time and financial cost associated with participatory approaches can be significant; and it is not always possible to compensate refugees/IDPs for their time [66]. Even if researchers intend to promote participation, refugees/IDPs may not always be accustomed to or comfortable with participating and may not engage as much as hoped [95]. Efforts to enable participants to co-create outputs may not always be successful as participants may be not used to having more autonomy and voice [67]. These challenges complicate efforts to promote refugee/IDP participation.

### Impacts of participation of refugees/IDPs in research

Some studies explicitly commented on the impacts of using participatory methods and strategies. For example, studies stated that using this approach to research increased participants’ well-being and confidence [55, 94]. Participants reported feeling heard [64]. Participatory research also created opportunities for socialisation amongst participants [73]. Engaging communities throughout the research enabled communities to create knowledge and develop local strategies for change [66].

Other studies did not specifically comment on concrete impacts but discussed the potential of participatory methods and strategies to contribute towards increasing solidarity [95], creating transformative experiences for participants [74], preventing research fatigue [95], and improving research rigour and ethics [69].

## Conclusion

This scoping review explored how the concept of participation is operationalised in research with refugees and IDPs. Our review highlights how despite recognition that participation of refugees/IDPs is important for research, the concept of participation continues to be used tokenistically, as a ‘buzzword’ [98] that is misappropriated to describe a myriad of research approaches and methods.

In our study, we found that while many studies use gender (including specifically drawing on feminist theory), or refugee/IDP status to explain the reason for taking a participatory approach, in many cases there was not a concerted effort to understand and outline the reasons why participation is important—and even less effort to document the impacts of using participatory approaches and methods. The power hierarchies within research more generally do provide a strong incentive for researchers to tackle imbalances inherent within the research process, however these dynamics were not often discussed in included papers. We suggest that conducting power analysis more broadly—including analysing power dynamics within research, gendered power dynamics and dynamics between refugess/IDPs and researchers—may provide stronger rationale for promoting participation, making it easier to identify concrete opportunities for refugee/IDP participation in research.

While only a small number of studies were classified as having limited/passing references to being participatory, those that did include references to either using participatory methods or participatory approaches more broadly, at times did not fully explain what exactly was participatory about the research. Methods like FGDs were described as being participatory, without it being clear what made this approach participatory. Even when approaches like CBPR or PAR were referenced, the descriptions of research practices were sometimes limited. Some of this gap is due to journal and peer reviewer expectations, as well as practices within research disciplines—rather than necessarily reflecting that participatory methods and approaches are not being used. Thus, we recommend more robust descriptions of how researchers action participation within research outputs, so that the wider research community can learn not only what they have accomplished, but how they accomplished it.

Where participatory approaches were used, we found that the use of specific strategies to promote participation tended to focus on involving refugees/IDPs in providing advice across the research process—a positive sign. In some cases, refugees were engaged as ‘peer researchers,’ though this strategy has also been critiqued by others as containing potential for exploitation [26, 99]. Importantly, engaging refugees/IDPs during analysis was less common, representing a gap in current strategies to promote participation, which others have also identified [100]. Thus, we suggest aiming to involve refugees and IDPs more in analysis, all whilst recognising also the additional burden on this engagement might place on refugees by seeking to find less time-intensive ways of seeking input on the findings and ensuring renumeration for this participation. Moreover, providing some kind of incentive or benefit for refugees/IDPs to participate was only mentioned in a few studies, although this would have meaningful impact for refugees/IDPs. While this review highlights that among refugees and IDPs there are limited examples of the systematic use of both participatory approaches across a research process, and use of participatory methods, we suggest much can be learnt from feminist participatory research among other populations. Feminist participatory research continues to provide innovative ways of understanding power, challenging how knowledge is produced (and by whom) and framing issues from women’s perspective [33, 36, 43]. However, many of these methodological advancements are yet to be tested in settings with refugees and IDPs. We suggest that particularly in humanitarian emergencies, the default assumption may be that using innovative methods is less realistic. Indeed, the urgent nature of the humanitarian response has at times acted as a justification for not considering issues of power in sufficient depth or not spending enough time to understand issues before responding [28, 101]. In the same way, the limited level of innovation within research methods among refugees and IDPs may be driven by assumptions about what is possible to implement within a humanitarian emergency. Notwithstanding the challenges in obtaining research funding for research in humanitarian settings that uses innovative methods, we suggest more work needs to be done to consider the value of participatory methods—beyond PhotoVoice—for research among refugees and IDPs.

We recommend that future research among refugees and IDPs should:

1. More explicitly detail how researchers sought to promote participation of refugees/IDPs, including clearer conceptualisations of what constitutes refugee/IDP participation and how they operationalised this.
2. Consider the use of innovative, feminist research methods that can challenge power dynamics and provide new opportunities for refugees and IDPs to share their lived experience. Learning from feminist participatory research methods used outside of refugee and IDP populations may provide important lessons to bring innovative research methods into the humanitarian sector.
3. Continue to engage refugees and IDPs in research design and analysis in particular, and use other strategies such as in-kind and financial compensation to recognise the contribution refugees and IDPs make towards research.
4. Include more explicit reflection on how power affects the research process and deliberately incorporate participatory approaches and methods to address this, including drawing on feminist and participation frameworks applied in other settings to ensure refugee/IDP participation is meaningful and not solely lip service. This should include consideration of how participatory approaches and methods align with key principles of rigorous, ethical research.
5. Seek to analyse the impacts of incorporating participatory approaches and methods on refugees/IDPs themselves, to help with documenting both positive impacts and unintended/negative impacts.

## Data Availability

All data generated or analysed during this study are included in this published article and its supplementary information files.
