# Correction: Participatory approaches and methods in gender equality and gender‑based violence research with refugees and internally displaced populations: a scoping review

**DOI:** 10.1186/s13031-023-00559-0

**Published:** 2024-01-03

**Authors:** Michelle Lokot, Erin Hartman, Iram Hashmi

**Affiliations:** London School of Hygiene and Tropical Medicine, London, UK

**Correction: Conflict and Health** 10.1186/s13031-023-00554-5 **.**

Following publication of the original article [1], the authors identified an error in caption of the Table 1. The correct Table 1 caption is given below.

Table 1 "Key search terms for each database".

The original article (1) has been corrected.
